# Experience and attitudes of pharmacists towards challenges and adaptive measures to new norm in ward pharmacy practice during the COVID-19 pandemic

**DOI:** 10.1186/s40545-023-00579-4

**Published:** 2023-07-10

**Authors:** Chew Beng Ng, You Leng Tan, Ros Sakinah Kamaludin, Chee Tao Chang, Chii-Chii Chew, Wai Keng Foong, Siew Huang Lee, Normi Hamdan, Su Yin Ong

**Affiliations:** 1 Pharmacy Department, Hospital Taiping, Ministry of Health Malaysia, Taiping, Malaysia; 2 Pharmacy Department, Hospital Raja Permaisuri Bainun, Ipoh, Ministry of Health Malaysia, Ipoh, Malaysia; 3 Clinical Research Centre, Hospital Raja Permaisuri Bainun, Ministry of Health Malaysia, Ipoh, Malaysia; 4 School of Pharmacy, Monash University Malaysia, Subang Jaya, Malaysia; 5 Pharmacy Department, Hospital Batu Gajah, Ministry of Health Malaysia, Batu Gajah, Malaysia; 6 Pharmacy Department, Hospital Kuala Kangsar, Ministry of Health Malaysia, Kuala Kangsar, Malaysia; 7 Pharmacy Department, Hospital Seri Manjung, Ministry of Health Malaysia, Seri Manjung, Malaysia; 8 Perak Pharmaceutical Services Division, Ministry of Health Malaysia, Tanjung Rambutan, Malaysia

**Keywords:** Challenges, Adaptive measure, Ward pharmacist, New norm, COVID-19 pandemic

## Abstract

**Background:**

COVID-19 pandemic has created challenges to the ward pharmacy practice. Challenges arose due to new norms in the ward pharmacy practice. Adaptive measures to overcome these challenges were important to sustain the quality of pharmaceutical care. This study aimed to identify the perceived challenges and attitudes towards adaptive measures in the ward pharmacy practice during the COVID-19 pandemic and determined their association with pharmacists’ characteristics.

**Method:**

This cross-sectional study was conducted in 14 Perak state hospitals and 12 primary health clinics through an online survey. All ward pharmacists and trainee pharmacists with at least 1 month of ward pharmacy experience and working in government-funded health facilities were included. The validated survey tool consisted of demographic characteristics, pharmacists’ experience towards challenges (22 items), and their attitude towards adaptive measures (9 items). Each item was measured based on a 5-point Likert scale. One-way ANOVA and logistic regression were employed to determine the association of pharmacists’ characteristics against their experience and attitude.

**Results:**

Out of 175 respondents, 144 (81.8%) were female, and 84 (47.7%) were Chinese. Most pharmacists served in the medical ward (124, 70.5%). Commonly reported perceived challenges were difficulties in counselling medication devices (3.63 ± 1.06), difficulties in clerking medication history from family members (3.63 ± 0.99), contacting family members (3.46 ± 0.90), patient’s digital illiteracy in virtual counselling (3.43 ± 1.11) and completeness of the electronic records (3.36 ± 0.99). For attitude towards adaptive measures, improving internet connection (4.62 ± 0.58), ensuring availability of multilingual counselling videos (4.45 ± 0.64), and provision of internet-enabled mobile devices (4.39 ± 0.76) were the most agreeable by the pharmacists. Male (AOR: 2.63, CI 1.12–6.16, p = 0.026) and master’s degree holders (AOR: 2.79, CI 0.95–8.25, p = 0.063) had greater odds of high perceived challenging experience scores. Master’s degree holders (AOR: 8.56, CI 1.741–42.069, p = 0.008) were also more likely to have a positive attitude score towards adaptive measures.

**Conclusion:**

Pharmacists faced multiple challenges in the ward pharmacy practice during the COVID-19 pandemic, especially in medication history assessment and patient counselling. Pharmacists, especially those with higher levels of education and longer tenure, exhibited a higher level of agreement towards the adaptive measures. The positive attitudes of pharmacists towards various adaptive measures, such as improvement of internet infrastructure and digital health literacy among patients and family members, warrant immediate action plans from health authorities.

## Introduction

The declaration of the COVID-19 pandemic, a global health emergency, on March 11, 2020, by the World Health Organization (WHO), had a tremendous impact on healthcare services, including pharmacy [1]. Globally, the COVID-19 pandemic had changed work practices for pharmacists, with many working overtime, experiencing an increased workload, and working with reduced staffing [2].

In Malaysia, pharmacy activities such as medication dispensing to out-patients and pharmacist-led medication therapy adherence clinics, which involved medication knowledge assessment, adherence evaluation, medication-related problem assessment, and therapeutic recommendations, were impacted by the government's quarantine policy and movement control order, leading to disruptions in appointment scheduling [3]. Extended pharmacy services such as drive-through pharmacies and drug supply via postal delivery were intensified in out-patient pharmacies to ensure continuity of medication supply and pharmaceutical care to patients [3, 4]. Temporary deployment of some ward pharmacists to the out-patient pharmacies to help with value-added service might also cause a shortage of pharmacists in the ward, affecting the ward pharmacy services [5]. Ward pharmacy services were hampered due to significant disruption in pharmaceutical care delivery in the ward [5–7], partly caused by tightened infection control practices.

The routine local ward pharmacy activities usually include medication history taking & reconciliation, case clerking, pharmacotherapy rounds with physicians, medication reviewing, medication counselling, and discharge planning for warded patients [8]. During the COVID-19 outbreak, visitor restriction policy, frequent changes of the medical wards into COVID-19 isolation wards and vice versa, and quarantine policies posed different challenges to ward pharmacy services. The usual ward pharmacy routine was changed to adapt to the “new norm”, i.e. to avoid 3Cs (crowded places, confined spaces, and close conversation) and to practice 3Ws (wash, wear and warn) [3, 10]. Pharmacists were split into teams to work on an alternate scheduling system to allow physical distancing and less crowding in the workplace [5, 9]. To limit contact with COVID-19 patients and to prevent unnecessary use of Personal Protective Equipment (PPE), remote inpatient review was conducted by clerking patients at pharmacy workstations instead of traditionally in the wards. This might affect counselling, medication review process, and participation in clinical rounds, impacting the quality of pharmaceutical care.

In the local settings, ward pharmacists implemented telepharmacy measures such as maintaining regular communication through phone calls or text messages with the patient care team. This facilitated streamlined drug information provision and remote screening of medication charts [5]. Pharmacists went the extra mile in patient counselling by creating instructional videos demonstrating techniques for using medical devices [5]. Ward pharmacists used phone or video call communication to perform virtual counselling and obtain patients' medication history, ensuring comprehensive pharmaceutical care [5, 9].

Current literature focused on the overall challenges and the adaptive strategies in pharmacy services during the COVID-19 pandemic [2–7, 9, 11–14]. The challenges the ward pharmacists encounter during COVID-19, and their attitude towards the adaptive measures have yet to be extensively explored. PPE and social distancing restrictions may present difficulties for pharmacists in delivering pharmaceutical care in the wards [5]. To conduct virtual counselling, pharmacists must have access to internet-enabled devices and a reliable internet connection. However, these resources may not be readily available in rural hospital settings. Pharmacists also encountered obstacles in delivering virtual counselling to patients with limited digital literacy or proficiency in operating electronic devices, particularly those from disadvantaged socioeconomic backgrounds who may not have access to mobile devices [12].

Understanding the perception and attitudes of pharmacists towards challenges and adaptive measures during the COVID-19 pandemic is crucial for maintaining quality patient care. It helps identify system barriers, provides targeted support, and informs training and resource allocation. Additionally, it contributes to understanding healthcare system resilience and preparedness for future crises.

Therefore, this study aimed to explore ward pharmacists' experiences in terms of challenges in the ward pharmacy practice according to new norms during the COVID-19 pandemic, their attitude towards possible adaptive measures to overcome these new challenges, and the associated characteristics.

## Methods

### Study design

This questionnaire-based, cross-sectional survey included all ward pharmacists and pharmacist trainees working in government-funded health facilities (14 hospitals and 12 primary health clinics) in Perak, a northwest state of peninsula Malaysia.

### Sampling and sample size

The total number of ward pharmacists was estimated based on internal administrative data provided by the Perak Pharmaceutical Services Division. With an estimated population of 200 ward pharmacists, a confidence interval of 95%, a margin of error of 5%, and a proportion of 50% of pharmacists with positive experiences and attitudes towards challenges and adaptive measures, 132 participants were needed for this study. The projected sample size of respondents was 147 after considering an estimation of a 90% completion rate (Raosoft sample size calculator). All pharmacists who fulfilled the eligibility criteria were sampled through convenience sampling.

### Inclusion and exclusion

We included pharmacists who met the following criteria: contract or permanently employed; had experience in the ward pharmacy practice for at least 1 month from January 1, 2021, onward; both trainees and fully registered pharmacists (FRP); served in the government-funded health facilities in Perak State; and consented to participate in the study. We excluded pharmacists who worked at mental health institutions.

### Instrument

The self-administered questionnaire was developed based on several studies following extensive literature reviews [5–7, 9, 12, 15]. The final version of the questionnaire consisted of three major domains: the demographic profile of respondents, the experience of pharmacists with perceived challenges (22 items), and the attitude of pharmacists towards adaptive measures (9 items). The questionnaire underwent face and content validation by two experts in the ward pharmacy. It was pre-tested on 10 respondents to ensure clarity. Then, a pilot test was conducted on 30 respondents to determine their reliability. The reliability of the two primary domains was demonstrated through satisfactory Cronbach’s alpha value (α) of more than 0.7: experience domain (α = 0.900); attitude domain (α = 0.793).

The questionnaire was developed into an online, electronic-based survey using Google Forms. With the assistance of the Perak Pharmaceutical Services Division, the link (URL) to the questionnaire was distributed to all chief pharmacists in the state via email and WhatsApp Messenger. The chief pharmacists then distributed the questionnaire to the potential participants in their facilities. Prior to accessing the questionnaire, the participants were directed to an electronic participant information sheet and an informed consent page. The consent form required respondents to choose their intention of participation. A "thank you" note would appear if they chose not to participate. Should they choose to participate, they clicked on “agree to participate” and answered the online questionnaire.

### Data analysis

The data were managed with SPSS version 20.0. The respondents’ sociodemographic characteristics were analysed descriptively. For perceived challenges scores, 1 point was given to “never”, and 5 points to “always”. The overall mean perceived challenges score was calculated by summing all the items in the perceived challenges domain and dividing by 22. The overall mean perceived challenges scores were then dichotomized, where a mean score equal to or more than 3.00 was categorized as high, and a mean score of less than 3.00 was categorized as low. The cut-off was decided based on the mean perceived challenging experience scores of 3.00 [16].

For attitude scores, 1 point was given for “strongly disagree” and 5 points for “strongly agree”. The overall mean attitude score was calculated by summing all the items in the attitude domain and dividing by 9. The overall mean attitude scores were then dichotomised, with a mean score of 4.00 or higher categorised as a positive attitude and a mean score of less than 4.00 categorised as a negative attitude. The cut-off was decided based on the mean attitude score of 4.00 [16].

One-way ANOVA analysis was performed to generate the mean scores of the perceived challenging experience and attitude domains for each socio-demographic characteristic. Univariate logistic regression was performed, and variables with a P-value less than 0.25 were included to run the multivariate logistic regression. Multicollinearity and interaction terms were checked and not found. The Hosmer–Lemeshow test, classification table and Nagelkerke R square were applied to check model fitness. Histogram and Kolmogorov–Smirnov test were performed to check normality. A backward stepwise multiple logistic regression model was used, and the final regression model included variables with p < 0.05. A Pearson correlation was used to determine the correlation between perceived challenging experience and attitude scores.

## Results

A total of 175 pharmacists out of 200 invited pharmacists responded. The majority were female (82.3%), between the ages of 21 and 30 (60.0%), Chinese (48.0%), had a bachelor's degree (89.1%), worked in medical wards (70.8%), and in non-COVID-19 wards (86.4%) (Table 1).

**Table 1:** Demographic characteristic of respondents with perceived challenges and attitudes score (n = 175)

Demographic	n = 175, n (%)	Perceived challenges		Attitude	
		Mean(SD)	p-value	Mean(SD)	p-value
Gender					
Male	31 (17.7%)	3.27 (0.53)	0.012*	4.16 (0.42)	0.180
Female	144 (82.3%)	2.95 (0.66)		4.27 (0.39)	
Ethnicity					
Malay	72 (41.1%)	2.85 (0.66)	0.010*	4.17 (0.43)	0.991
Chinese	84 (48.0%)	3.15 (0.56)		4.18 (0.40)	
Indian	19 (10.9%)	2.94 (0.83)		4.18 (0.45)	
Age group					
21–30 years old	105 (60.0%)	2.96 (0.67)	0.258	4.14 (0.39)	0.216
31–50 years old	70 (40.0%)	3.07 (0.61)		4.22 (0.44)	
Years of service					
< 1 year	60 (34.3%)	2.80 (0.68)	0.025*	4.16 (0.39)	0.995
1–5 years	78 (44.6%)	3.09 (0.64)		4.18 (0.44)	
6–10 years	22 (12.6%)	3.19 (0.54)		4.19 (0.37)	
11–15 years	15 (8.6%)	3.11 (0.53)		4.18 (0.46)	
Employment type					
Trainee	56 (32.0%)	2.79 (0.63)	0.009*	4.10 (0.40)	0.044*
Contract-based FRP	26 (14.9%)	3.01 (0.62)		4.07 (0.40)	
Permanent-based FRP	93 (53.1%)	3.13 (0.64)		4.25 (0.41)	
Highest education level					
Bachelor’s Degree	156 (89.1%)	2.97 (0.66)	0.047*	4.16 (0.41)	0.089
Master’s Degree	19 (10.9%)	3.28 (0.55)		4.33 (0.43)	
Wad discipline					
Medical	124 (70.9%)	2.99 (0.66)	0.800	4.16 (0.42)	0.468
Pediatrics	18 (10.3%)	2.87 (0.68)		4.07 (0.46)	
Surgical	9 (5.1%)	3.22 (0.67)		4.41 (0.49)	
Orthopedics	6 (3.4%)	3.20 (0.48)		4.28 (0.21)	
ICU	5 (2.9%)	2.87 (0.70)		4.36 (0.14)	
A&E	3(1.7%)	3.26 (0.31)		4.04 (0.17)	
Multidisciplinary	6 (3.4%)	2.88 (0.71)		4.31 (0.36)	
Others	4 (2.3%)	3.27 (0.41)		4.22 (0.33)	
Ward type					
Non-COVID-19	152 (86.9%)	3.02 (0.67)	0.322	4.17 (0.42)	0.727
COVID-19 and non-COVID-19	23 (13.1%)	2.88 (0.44)		4.20 (0.38)	
PhIS type					
Pharmacy based	109 (62.3%)	2.92 (0.60)	0.168	4.07 (0.44)	0.007*
Full based	66 (37.7%)	3.06 (0.67)		4.24 (0.39)	

PRP: Provisionally registered pharmacist; FRP: Fully registered pharmacist; HDU: High-dependency care unit; PhIS: Pharmacy Information System; SD: standard deviation

Full based: Medication prescribed by doctors was directly entered into the PhIS system

Pharmacy based: Pharmacists manually transcribed prescriptions written by doctors into the PhIS system

^*^One-way ANOVA test performed (Independent t-test performed if 2 categories variables)

Out of a maximum score of 5, the overall mean perceived challenges score was 3.00 ± 0.65, ranging from 1.14 to 4.64. The pharmacists most reported perceived challenges in the following areas: difficulties in counselling certain medication devices (3.63 ± 1.06), difficulties in clerking medication histories from family members (3.63 ± 0.99), difficulties in contacting family members (3.46 ± 0.90), patient’s digital illiteracy in virtual counselling (3.43 ± 1.11) and completeness of the electronic health records (3.36 ± 0.99) (Table 2).

**Table 2:** Perceived challenges by pharmacists during COVID-19 pandemic in wards

No	Challenges	Never n (%)	Rarely n (%)	Occasionally n (%)	Often n (%)	Always n (%)	Mean (SD)
1	Insufficient personal protective equipment (e.g.: face shield, surgical mask, N95 mask)	40 (22.9)	53 (30.3)	55 (31.4)	21 (12.0)	6 (3.4)	2.43 (1.07)
2	Shortage of staff from time to time due to quarantine	8 (4.6)	38 (21.7)	64 (36.6)	50 (28.6)	15 (8.6)	3.15 (1.01)
3	Inadequate medications for COVID-19 treatment	35 (20.0)	67 (38.3)	46 (26.3)	21 (12.0)	6 (3.4)	2.41 (1.05)
4	Frequent drug shortages for patient’s treatment	17 (9.7)	71 (40.6)	49 (28.0)	24 (13.7)	14 (8.0)	2.69 (1.08)
5	Frequent conversion of ward to manage Covid-19 patients (eg: covid ward to non-covid ward and vice versa)	19 (10.9)	28 (16.0)	62 (35.4)	52 (29.7)	14 (8.0)	3.08 (1.10)
6	Frequent changes in COVID-19 updates from the hospital management	10 (5.7)	42 (24.0)	70 (40.0)	44 (25.1)	9 (5.1)	3.00 (0.96)
7	The off-label and compassionate use of novel experimental agents	30 (17.1)	64 (36.6)	62 (35.4)	18 (10.3)	1 (0.6)	2.41 (0.91)
8	Limited internet connection to provide ward pharmacy service virtually	25 (14.3)	32 (18.3)	40 (22.9)	43 (24.6)	35 (20.0)	3.18 (1.33)
9	Difficulties in tracing patients’ own medication history within 24–48 h	10 (5.7)	39 (22.3)	72 (41.1)	37 (21.1)	17 (9.7)	3.07 (1.03)
10	Difficulties in reaching patients’ family members for medication reconciliation (family member not allowed to visit / patient is poor historian)	1 (0.6)	22 (12.6)	71 (40.6)	57 (32.6)	24 (13.7)	3.46 (0.90)
11	Family member unable to inform the patient’s medication list through phone as unsure about the name of medication	4 (2.3)	19 (10.9)	49 (28.0)	69 (39.4)	34 (19.4)	3.63 (0.99)
12	Communication barriers with patients or family members when wearing face masks and face shield	16 (9.1)	48 (27.4)	52 (29.7)	45 (25.7)	14 (8.0)	2.96 (1.11)
13	Communication barriers with patients or family members due to social distance	20 (11.4)	42 (24.0)	63 (36.0)	40 (22.9)	10 (5.7)	2.87 (1.07)
14	Delay in medication history clerking due to pending COVID-19 tests result	19 (10.9)	44 (25.1)	50 (28.6)	46 (26.3)	16 (9.1)	2.98 (1.15)
15	Difficulties to interview patient for medication history within 15 min (reduce patient contact time)	10 (5.7)	51 (29.1)	56 (32.0)	34 (19.4)	24 (13.7)	3.06 (1.13)
16	Uncertain about the accuracy and completeness of patients’ medication histories traced solely from PhIS system	5 (2.9)	29 (16.6)	61 (34.9)	58 (33.1)	22 (12.6)	3.36 (0.99)
17	Limited assessment of patients in the ward with no participation in clinical rounds with the physicians	24 (13.7)	71 (40.6)	42 (24.0)	31 (17.7)	7 (4.0)	2.58 (1.06)
18	Difficulties to discuss with prescriber for pharmacotherapy plan of patient	24 (13.7)	83 (47.4)	46 (26.3)	16 (9.1)	6 (3.4)	2.41 (0.95)
19	Patient do not own smartphone to assess virtual counselling	9 (5.1)	41 (23.4)	58 (33.1)	52 (29.7)	15 (8.6)	3.13 (1.03)
20	Patient’s digital illiteracy causing difficulties in virtual counselling	10 (5.7)	26 (14.9)	48 (27.4)	61 (34.9)	30 (17.1)	3.43 (1.11)
21	Difficulties in counselling certain medication devices (eg: difficult to assess MDI technique due to face mask wearing)	7 (4.0)	17 (9.7)	48 (27.4)	64 (36.6)	39 (22.3)	3.63 (1.06)
22	Patients had problems understanding virtual counselling videos due to language barriers	13 (7.4)	31 (17.7)	62 (35.4)	51 (29.1)	18 (10.3)	3.17 (1.07)

Regarding attitudes toward adaptive measures, the overall mean score was 4.00 ± 0.38 out of a maximum of 5, ranging from 2.89 to 4.78. The pharmacists were more agreeable to such adaptive measures: improving internet connection (4.62 ± 0.58), ensuring availability of multilingual counselling videos (4.45 ± 0.64), providing internet-enabled mobile devices (4.39 ± 0.76), educating physicians about formulary restrictions (4.25 ± 0.62), and developing local COVID-19 treatment (4.15 ± 0.65) (Table 3).

**Table 3:** Attitude of pharmacists towards adaptive measures during COVID-19 pandemic in wards

No	Adaptive measures	Strongly disagree n (%)	Disagree n (%)	Neutral n (%)	Agree n (%)	Strongly agree n (%)	Mean (SD)
1	Implement drug shortage mitigation strategies	1 (0.6)	2 (1.1)	61 (34.9)	82 (46.9)	29 (16.6)	3.78 (0.75)
2	Prioritize drug supply to patients who are most likely to benefit	2 (1.1)	2 (1.1)	24 (13.7)	97 (55.4)	50 (28.6)	4.09 (0.75)
3	Formulary restrictions for COVID-19 therapies should be developed (i.e. restrictions on hospital drug use due to limited supply)	1 (0.6)	7 (4.0)	40 (22.9)	99 (56.6)	28 (16.0)	3.83 (0.76)
4	Develop local COVID-19 treatment protocol	1 (0.6)	1 (0.6)	16 (9.1)	109 (62.3)	48 (27.4)	4.15 (0.65)
5	Educating physicians about formulary restrictions	0 (0.0)	2 (1.1)	11 (6.3)	103 (58.9)	59 (33.7)	4.25 (0.62)
6	Remote inpatient review via electronic Hospital Information System	1 (0.6)	3 (1.7)	43 (24.6)	77 (44.0)	51 (29.1)	3.99 (0.81)
7	Improvement of internet connection in hospital	0 (0.0)	0 (0.0)	9 (5.1)	48 (27.4)	118 (67.4)	4.62 (0.58)
8	Hospital to provide internet-enabled mobile devices for pharmacists to perform virtual counselling and medication history assessment	2 (1.1)	1 (0.6)	15 (8.6)	65 (37.1)	92 (52.6)	4.39 (0.76)
9	Availability of drug counselling videos in other languages besides Malay and English language	0 (0.0)	1 (0.6)	11 (6.3)	71 (40.6)	92 (52.6)	4.45 (0.64)

It was found that males (p = 0.012), Chinese (p = 0.010), possessing a master’s degree (p = 0.047) and permanent-based FRP (p = 0.009) were associated with higher perceived challenging experience scores. In contrast, pharmacists working in pharmacy-based information systems were associated with positive attitude scores (p = 0.010) (Table 4).

**Table 4:** Comparison of demographic characteristics with perceived challenges and attitudes score (n = 175)

Variable	Freq (n)	Perceived challenges		Attitudes	
		Mean (SD)	p-value	Mean (SD)	p-value
Age					
21–30	105	2.96 (0.67)	0.258	4.14 (0.39)	0.216
31–50	70	3.07 (0.61)		4.22 (0.44)	
Gender					
Male	31	3.27 (0.53)	0.012	4.16 (0.42)	0.180
Female	144	2.95 (0.66)		4.27 (0.39)	
Ethnicity					
Malay	72	2.85 (0.66)	0.010	4.17 (0.43)	0.991
Chinese	84	3.15 (0.56)		4.18 (0.40)	
Indian	19	2.94 (0.83)		4.18 (0.45)	
Education					
Bachelor degree	156	2.97 (0.66)	0.047	4.16 (0.41)	0.089
Master degree	19	3.28 (0.55)		4.33 (0.43)	
Employment type					
Permanent-based FRP	93	3.13 (0.64)	0.009	4.25 (0.41)	0.044
Contract-based FRP	26	3.01 (0.62)		4.07 (0.40)	
Trainee	56	2.79 (0.63)		4.10 (0.40)	
Wards covered					
COVID-19 & non COVID-19	23	2.88 (0.44)	0.322	4.20 (0.38)	0.727
Non COVID-19	152	3.02 (0.67)		4.17 (0.42)	
PhIS type					
Full based	66	2.92 (0.60)	0.168	4.07 (0.44)	0.007
Pharmacy based	109	3.06 (0.67)		4.24 (0.39)	
Discipline of wards					
Medical	124	2.99 (0.66)	0.800	4.16 (0.42)	0.468
Pediatrics	18	2.87 (0.68)		4.07 (0.46)	
Surgical	9	3.22 (0.67)		4.41 (0.49)	
Orthopedics	6	3.20 (0.48)		4.28 (0.21)	
ICU	5	2.87 (0.70)		4.36 (0.14)	
A&E	3	3.26 (0.31)		4.04 (0.17)	
Multidisciplinary	6	2.88 (0.71)		4.31 (0.36)	
Others	4	3.27 (0.41)		4.22 (0.33)	
Duration of service					
< 1 year	60	2.80 (0.68)	0.025	4.16 (0.39)	0.995
1–5 years	78	3.09 (0.64)		4.18 (0.44)	
6–10 years	22	3.19 (0.54)		4.19 (0.37)	
11–15 years	15	3.11 (0.53)		4.18 (0.46)	
Overall		3.00 (0.65)		4.17 (0.41)	

One-way ANOVA was performed. PhIS: Pharmacy Information System Full based: Medication prescribed by doctors was directly entered into the PhIS system. Pharmacy based: Pharmacists manually transcribed prescriptions written by doctors into the PhIS system

The male pharmacists (AOR: 2.63, CI 1.12–6.16, p = 0.026) and those with a master’s degree (AOR: 2.79, CI 0.95–8.25, p = 0.063) had higher odds of perceived challenging experience scores. Pharmacists with a master’s degree (AOR: 8.56, CI 1.74–42.07, p = 0.008), contract-based FRP (AOR: 3.21, CI 1.30–7.94, p = 0.012), permanent-based FRP (AOR: 2.92, CI 1.03–8.33, p = 0.045), those who worked in pharmacy-based information system (AOR: 2.57, CI 1.32–4.99, p = 0.006) and those who worked for more than 1 year were more likely to have a positive attitude towards adaptive measures (Tables 5 and 6).

**Table 5:** Univariate logistics regression for significant factors associated with perceived challenging experience and attitude scores (n = 175)

Variable	Perceived challenging experience scores			Attitudes scores		
	Unadjusted OR (β)	Confidence interval	P	Unadjusted OR (β)	Confidence interval	P
Age						
21–30	Ref			Ref		
31–50	0.682	0.371–1.253	0.218	0.981	0.534–1.803	0.950
Gender						
Female	Ref			Ref		
Male	2.732	1.177–6.339	0.019	1.582	0.707–3.540	0.264
Ethnicity						
Malay	Ref			Ref		
Chinese	1.667	0.884–3.144	0.115	1.556	0.564–4.293	0.394
Indian	1.200	0.442–3.258	0.721	1.157	0.612–2.184	0.654
Education						
Bachelor’s degree	Ref			Ref		
Master’s degree	2.947	1.013–8.578	0.047	3.384	1.075–10.654	0.037
Employment type						
Trainee	Ref			Ref		
Contract-based FRP	1.385	0.579–3.312	0.465	1.701	0.870–3.323	0.120
Permanent-based FRP	1.987	1.013–3.894	0.046	1.583	0.660–3.797	0.303
Wards covered						
COVID-19 &Non COVID-19	Ref			Ref		
Non COVID-19	1.444	0.597–3.495	0.415	0.860	0.357–2.070	0.736
PhIS type						
Full-based	Ref			Ref		
Pharmacy based	1.208	0.655–2.228	0.544	2.341	1.254–4.371	0.008
Duration of service						
< 1 year	Ref			Ref		
1–5 years	0.875	0.446–1.717	0.698	1.452	0.732–2.878	0.286
6–10 years	0.408	0.146–1.144	0.088	1.930	0.719–5.182	0.192
11–15 years	0.766	0.246–2.380	0.644	1.072	0.337–3.410	0.906

PhIS: Pharmacy Information System. Full based: Medication prescribed by doctors was directly entered into the PhIS system. Pharmacy based: Pharmacists manually transcribed prescriptions written by doctors into the PhIS system

**Table 6:** Multivariate logistics regression for significant factors associated with perceived challenging experience and attitude scores (n = 175)

Variable	Perceived challenging experience scores			Attitudes scores		
	Nagelkerke R^2^ = 0.072, Hosmer and Lemeshow test = 0.897, classification table = 59.4%			Nagelkerke R^2^ = 0.192, Hosmer and Lemeshow test = 0.336, classification table = 64.0%		
	Adjusted odd ratios (β)	Confidence interval	P	Adjusted odd ratios (β)	Confidence interval	P
Age						
21–30						
31–50						
Gender						
Female	Ref					
Male	2.632	1.124–6.161	0.026			
Ethnicity						
Malay						
Chinese						
Indian						
Education						
Bachelor’s degree	Ref			Ref		
Master’s degree	2.792	0.945–8.246	0.063	8.558	1.741–42.069	0.008
Employment type						
Trainee				Ref		
Contract-based FRP				3.209	1.297–7.937	0.012
Permanent-based FRP				2.923	1.026–8.325	0.045
Wards covered						
COVID-19 & non COVID-19						
Non COVID-19						
PhIS type						
Full based				Ref		
Pharmacy based				2.565	1.318–4.990	0.006
Duration of service						
< 1 year				Ref		
1–5 years				2.623	1.133–6.073	0.024
6–10 years				9.553	2.391–38.166	0.001
11–15 years				11.861	2.014–69.847	0.006

Backward stepwise multiple logistic regression analysis. FRP: Fully registered pharmacist. PhIS: Pharmacy Information System. Full based: Medication prescribed by doctors was directly entered into the PhIS system. Pharmacy based: Pharmacists manually transcribed prescriptions written by doctors into the PhIS system

There was a significant, positive, moderate correlation between perceived challenges and attitude scores (Pearson correlation: 0.332, p < 0.001).

## Discussion

To the best of our knowledge, this was the first study that assessed the perception of ward pharmacists towards challenges and adaptive measures implemented in the wards. The respondents reported different levels of challenges in carrying out ward pharmacy duties. The highest-ranked perceived challenge was medical device counselling, consistent with the findings of other studies [5, 9, 12, 17]. Social distancing, personal protective equipment (PPE), and the restriction of pharmacist entry into the ward during the COVID-19 pandemic introduced significant communication barriers between pharmacists and patients [5, 9, 12, 17]. It hinders pharmacists' ability to demonstrate and reassess medical devices' use effectively. Patients who used medical devices such as inhalers or insulin pens required continuous patient education and constant assessment of their technique [17]. The challenges could be overcome by introducing telepharmacy services and providing counselling virtually [9, 12, 18]. However, patients with low digital literacy, older age, lower socioeconomic status, and no electronic devices might not benefit from this innovative strategy [12, 18–20]. Hence, remote devices like smartphones or intercom systems in the wards could be a feasible alternative [9].

Malaysia is a multiracial nation, and language barriers could present in patient communication when providing medication counselling [21]. Ward pharmacists could create multi-lingual videos to help patients and caregivers learn medical device usage in their preferred languages [22]. This was in line with the evidence that providing educational materials in patients' preferred languages improved their health literacy [23, 24]. Therefore, creating multi-lingual instruction videos for medical device counselling could benefit a multiracial population.

The second highest-rated challenge was obtaining a comprehensive medication list from family members. Communication with family members was required when patients had low health literacy and language barriers to providing medication information. Face-to-face communication between pharmacists and the patients' family members was restricted because of the "no visitor policy" during the pandemic. Furthermore, due to home quarantine, the patient's family member might be unable to deliver the patient's previous drugs to the ward [9]. Thus, communication could only be done via telephone, which increased the risk of misinformation and medication errors [22].

The Pharmacy Information System (PhIS) was an electronic prescribing and medication supply record system widely used in Malaysian government-funded hospitals [12]. Tracing patient medication records from PhIS to obtain a complete medication history was common during the pandemic. Similarly, the use of secondary sources of information via a database in completing medication histories was previously reported [22]. However, the respondents recognized that improving the accuracy and completeness of patient medication history in the PhIS could enhance the quality of pharmaceutical care provided. These findings suggested the importance of regular updates to patient prescription data and the inclusion of more comprehensive information regarding the use of over-the-counter (OTC) and herbal supplements within the PhIS system [22]. However, implementing electronic medical records could lead to increased financial and time costs, as physicians might need to dedicate more time to managing and updating medical records [26]. The feasibility of electronic medical records (EMR) [9], allowing one visitor during a restricted time window, and taking the history via video conferencing [17] should be studied to aid medication history clerking during the pandemic.

Digital health insufficiency might affect the delivery of pharmaceutical care. Similar to our study, internet connectivity issues and a lack of suitable platforms for performing telepharmacy was reported [27–29]. High internet bandwidth and proper equipment were required to ensure good audio and visual quality during video conferencing [30, 31]. Nevertheless, a secure platform explicitly designed to protect patient privacy and adhere to the regulations was needed [19]. For example, APOmondo was a free telepharmacy portal created in Germany to provide personal care to patients [32]. However, the initial investment in developing and operational costs of maintaining the telepharmacy infrastructure could be substantial, especially for lower and middle-income countries [33]. Hence, the stakeholders should assess the viability of different available options to increase the accessibility of telepharmacy services [12].

In the early phase of the pandemic, several medications were suggested to be repurposed for the treatment of COVID-19. However, the evidence supporting the use of these agents was inadequate due to a lack of safety and efficacy data [6, 7]. The development of local treatment protocols with formulary restrictions was an essential adaptive measure agreed upon by pharmacists in this study [6, 7, 9, 34]. These guidelines assisted clinicians and pharmacists in better understanding the treatment of COVID-19 patients [7] and alerting prescribers when medications are in limited supply and restricted for COVID-19 [6].

Insufficient personal protective equipment, shortage of staff and drug shortages were not perceived as the most common challenges by the respondents, but these were the top three pharmacists’ concerns according to a Canadian study [35]. Meanwhile, pharmacists listed mental health as the fourth most important concern [35]. Mental health problems might affect pharmacists’ capacity to make rational decisions, provide high-quality care, and impair quality of life and job satisfaction [36, 37]. Poor mental health could result in a shortage of pharmacists after the pandemic, causing serious implications for the health system. It was important for stakeholders to develop resilience by implementing early screening and detection of mental illness, quality case management and awareness programmes to prepare the health system for potential outbreaks in the future.

Our study found that male pharmacists and those with master's degrees had higher odds of perceiving challenging experiences. The "reality" of the job environment was influenced by perception, attitude, and opinion. All these factors might differ according to certain characteristics, such as gender [38] and the work experience of pharmacists in practice [39]. On the other hand, the literature review by Carvajal et al. reported that workplace challenges were received to an equal extent by both male and female pharmacists. Nevertheless, challenges, when taken as opportunities for advancement, could contribute to job satisfaction and the development of ward pharmacy services [39].

This research revealed that fully registered pharmacists, whether on a contract or permanent basis and pharmacists who had worked for more than a year, were more likely to have a positive attitude toward workplace adaptive measures during the pandemic. The pharmacist’s positive attitude towards providing pharmaceutical care was important in gaining patients' confidence in the profession [40]. Generally, pharmacists had positive attitudes towards their undertakings and interest in constantly improving pharmaceutical care services [40, 41]. Education level impacted the attitude of the pharmacist positively [42], in congruence with our finding that master's degree holders were more optimistic about adapting to the new norm in the ward. Positive attitudes might contribute to increased productivity [5]. Therefore, the new norms should not be perceived as a barrier to pharmacists' ability to provide effective pharmaceutical care.

### Study limitations

Our study was conducted only in the Perak state; hence the findings might not reflect the situation encountered in other states in this country. Furthermore, recall bias might be present among respondents who were no longer working in the ward when answering the survey. In addition, some ward pharmacists were transferred between facilities during the pandemic, causing difficulty in obtaining responses. Nevertheless, the state pharmaceutical service division's involvement maximised the questionnaire's dissemination.

## Conclusion

During the COVID-19 pandemic, multiple challenges arise in the ward pharmacists’ daily routine, particularly when assessing patients' prescription histories and providing patient counselling. Pharmacists demonstrated a high level of agreement towards the adaptive measures, particularly those with a higher level of education and longer duration of service. Health authorities should develop an early action plan in light of pharmacists' positive attitude towards adaptive measures, such as upgrading internet infrastructure and preparing the health system for digital health and telepharmacy.

## Data Availability

The datasets used and/or analysed during the current study available from the corresponding author on reasonable request.
